# Towards community-driven tuberculosis education: findings from a knowledge and engagement pilot survey in the rural community of Eastern Cape, South Africa

**DOI:** 10.3389/fpubh.2025.1671871

**Published:** 2025-11-19

**Authors:** Ntandazo Dlatu, Urgent Tsuro, Lindiwe Modest Faye, Mojisola Clara Hosu, Walter Sisulu University TB Research Group Medical Microbiology 2025 Honours Students, Bulela Sonka, Hloniphani Guma, Londiwe Nxumalo, Luvo Ntombana, Luzuko Mkono, Mandlenkosi Manika, Mbulelo Cebisa, Muziwabantu Joyi, Nande Ndamase, Phumelela Bana, Siphosihle Conham, Sithabile Moso, Yanga Somjobo, Sineke Ncomeka, Teke Apalata

**Affiliations:** 1 Department of Public Health, Faculty of Medicine and Health Sciences, Walter Sisulu University, Mthatha, South Africa; 2 Division of Medical Microbiology, Department of Laboratory-Medicine & Pathology, Faculty of Health Sciences, Walter Sisulu University, Mthatha, South Africa; 3 Department of Laboratory Medicine and Pathology, Faculty of Medicine and Health Sciences, Walter Sisulu University, Mthatha, South Africa

**Keywords:** tuberculosis (TB), TB knowledge, stigma, barriers to testing, rural South Africa, Eastern Cape, health education, KAP survey

## Abstract

**Background:**

Tuberculosis (TB) remains a major public health concern in rural South Africa, with widespread community knowledge gaps and pervasive stigma significantly impeding early diagnosis and treatment success. This pilot study evaluated TB knowledge and barriers to testing among community members in Ntabankulu, Eastern Cape, to inform targeted, community-driven education strategies.

**Methods:**

A cross-sectional survey utilizing a structured Knowledge-Attitudes-Practices (KAP-TB) questionnaire was administered to 131 rural community members. TB knowledge was categorized into low, moderate, and high levels based on scores derived from a Likert-type scale. Statistical analysis used Fisher’s exact and Kruskal–Wallis tests to examine associations between knowledge levels, sociodemographic variables, barriers, and TB exposure history. Boxplots provided visual insight into distributions across age and gender.

**Results:**

Among participants, TB knowledge was mostly moderate (64.9%), with 11.5% reporting low knowledge and 23.7% high knowledge. Knowledge was significantly associated with education level (*p* < 0.001): 52% of the high-knowledge group had a tertiary education, compared to none in the low-knowledge group. Although gender (*p* = 0.5) and age (*p* = 0.2) were not statistically significant overall, boxplot visualization suggested a trend toward higher knowledge scores among younger, male participants, especially those with a history of TB exposure. The most frequently cited barriers to testing were fear of stigma (42%) and lack of knowledge (33%). Low-knowledge participants more frequently reported structural barriers such as distance (10%) and cost (7%). Crucially, participants with a personal (*p* = 0.047) or family (*p* < 0.001) history of TB experience were significantly more likely to have high knowledge.

**Conclusion:**

TB knowledge in this rural setting is primarily shaped by formal education and direct personal experience, while stigma and misinformation remain the predominant barriers to timely testing. Future community-driven education must prioritize leveraging survivor storytelling, peer education, and culturally tailored messaging to simultaneously boost TB literacy, actively reduce stigma, and promote prompt care-seeking.

## Introduction

Tuberculosis (TB) remains a global public health emergency and is particularly devastating in rural, under-resourced regions. Despite biomedical advancements, the disease’s persistence is closely linked to socioeconomic determinants and limited community involvement in TB control strategies. The 2024 WHO Global Report highlights the need for integrated, people-centered interventions to end TB by 2035, especially in high-burden countries such as South Africa (1). South Africa continues to feature among the WHO’s high TB burden countries, with an incidence of approximately 468 per 100,000 population (1, 2). The Eastern Cape province carries a disproportionate burden of TB cases. Being one of South Africa’s poorest provinces, the fight against TB is complicated by poverty, limited healthcare access, stigma, and misinformation (3). Addressing these challenges requires not only improved diagnostics and treatments but also robust community engagement (4, 5). Community engagement (CE) is increasingly recognized as a critical pillar in public health research and implementation. It enables the development of interventions that are culturally sensitive, acceptable, and sustainable. CE in TB research has shifted from mere consultation to active collaboration. This encompasses active participation of affected communities, including patients, families, local leaders, and NGOs, in shaping and implementing interventions. Recent evidence highlights several key models and outcomes, including Community advisory boards (CABs), Community-based Participatory Research (CBPR), Structured Community Networks and Engagement, and facility-based Community Advisory groups. These models have improved research literacy and ensured community voices influence study design and policy, helped identify service delivery barriers and empower patients through co-learning and social support, reduced stigma and improved treatment adherence, facilitated trust and improved acceptability of new tools (6–8). Evidence from Ethiopia, Mexico, Peru, and Vietnam shows that CE improves TB knowledge, reduces stigma, enhances case detection, and builds trust between healthcare systems and communities (7–9). Yet, in many TB-endemic regions, CE remains underutilized. The current study builds on this global movement by assessing community knowledge, engagement levels, and barriers to TB testing in Ntabankulu, Eastern Cape. Our goal is to generate evidence that can guide context-appropriate, community-led education and engagement strategies, essential components of the WHO’s End TB Strategy and the UN’s High-Level Meeting on TB outcomes (10, 11). Understanding the knowledge, attitudes, and practices (KAP) related to TB among community members is crucial to the targeted development of TB prevention and control efforts (12). Inadequate knowledge about TB prevention will lead to delayed case detection and timely treatment initiation. Community-driven TB education is an essential strategy for improving awareness, reducing stigma, and encouraging early case detection, especially in settings where health services struggle to reach the most vulnerable. A study in rural KwaZulu-Natal revealed that community education significantly enhanced knowledge about TB and TB preventive therapy (TPT). The study opined that greater community-level public health education and individual-level counseling efforts are essential to facilitate TPT expansion and implementation (13); thus highlighting the power of local, context-sensitive education in improving community understanding. In Uganda, community health workers (CHWs) played a central role in active TB case finding and health promotion, reaching underserved rural populations. They not only identified more TB cases than formal systems but also provided culturally relevant TB education, thus encouraging testing and enhancing follow-up in hard-to-reach areas (14). This highlights the effectiveness of empowering community members as agents of health education. Community-driven TB education implemented through local health workers, participatory interventions, or community-led monitoring has been shown to significantly improve TB knowledge, reduce stigma, and enhance case detection. These approaches are particularly effective in rural, underserved settings, exemplifying scalable models for ending TB. Therefore, promoting a correct understanding of people and comprehensive knowledge of TB through increasing community mobilization and advocacy is highly essential. This pilot study aimed to assess TB knowledge, levels of community engagement with TB information and services, and barriers to testing among community members in Ntabankulu, Eastern Cape, to inform community-driven education strategies.

## Methods

### Research design

A cross-sectional research design was used. A KAP-TB questionnaire assessed participants’ *knowledge* (symptoms, transmission, and treatment of TB), *attitude*, and *health-seeking* behavior. The participants were those presenting at a multipurpose center in the Ntabankulu local municipality during a community health outreach. Knowledge scores were computed and categorized into low, moderate, and high levels for analysis.

### Study setting

The study was conducted in the rural Ntabankulu local municipality, located within the Alfred Nzo District of the Eastern Cape province. This area, which is home to an estimated 831,112 isiXhosa people, is the smallest of the four local municipalities in the district, covering 1,385 km^2^ (13% of the district’s geographical area). The name “Ntabankulu” is an isiXhosa term meaning “great mountain,” a fitting name given the area’s mountainous terrain. The main economic sectors are agriculture, forestry, fishing, mining and quarrying, manufacturing, electricity, and construction.

### Study participants

Patients aged 15 years and older who received various medical services at the community health outreach were invited to participate. In accordance with the National Health Act (No. 61 of 2003), written informed consent was obtained from parents or legal guardians for participants under 18. Additionally, participants aged 12–17 provided written assent. Self-reported ethnicity was categorized using standard South African demographic classifications. The term ‘Colored’ was used to match the official population group classifications in South Africa for demographic and health research.

### Sampling technique

A convenience sampling approach was employed to include participants who presented at the community health outreach for health consultations and treatment. Those who were willing to participate and consented to the study were included.

### Data collection and instruments

A structured KAP-TB questionnaire, adapted from a standardized World Health Organization (WHO) tool, was used to collect data on participants’ sociodemographic characteristics, as well as their knowledge, attitudes, and practices regarding tuberculosis. The practices section specifically focused on health-seeking behaviors and barriers to testing and care (15). Minor modifications were made to ensure the questionnaire was culturally appropriate for the rural Eastern Cape community.

The questionnaire was initially drafted in English and then translated into isiXhosa. To check for clarity and suitability, the isiXhosa version was pre-tested with a group of isiXhosa-speaking Medical Microbiology students. Although a formal Cronbach’s alpha analysis was not conducted for this pilot study, corrections were made to ensure language consistency.

### Participant recruitment and sampling

A convenience sampling approach was employed to recruit participants who were present at a community health outreach for various medical services. Due to the nature of this sampling method, individuals who were too unwell to attend the outreach were excluded by default. As this was a pilot study, a formal sample size calculation was not performed; a total of 131 participants were included.

Voluntary participation was emphasized, and all participants provided informed consent. The consent form explicitly stated that they could withdraw from the study at any time without their decision affecting their consultation at the outreach program.

### Data management

Researchers and trained data collectors assisted participants who had difficulty reading or writing. Completed questionnaires were double-checked to maintain data quality.

### Data analysis

Data was cleaned, checked for errors, and entered into an Excel spreadsheet for analysis using SPSS Statistics version 29. Descriptive statistics were used to summarize participants’ demographic characteristics with frequencies and percentages. A *p*-value of less than 0.05 was considered statistically significant. Participants’ knowledge scores, computed from their responses to the KAP-TB questionnaire, were categorized as low, moderate, or high. The associations between these knowledge levels and sociodemographic variables, barriers to testing, and TB exposure history were analyzed using Fisher’s exact test and the Kruskal–Wallis test. Participants’ TB knowledge was assessed using 12 structured questions covering symptoms, modes of transmission, prevention, and treatment of TB. Each question was scored on a binary scale (1 = correct, 0 = incorrect), giving a total possible score range of 0–12. For descriptive analysis, individual scores were converted into percentage scores. Knowledge levels were then categorized as follows, based on widely used cut-off points in KAP-TB surveys:

- *Low knowledge*: scores ≤ 40% (0–4 correct answers).
- *Moderate knowledge*: scores 41–70% (5–8 correct answers).
- *High knowledge*: scores ≥ 71% (9–12 correct answers).

### Ethics approval

This study was conducted by the principles of good scientific practice and was approved by the Ethics Committee of the Walter Sisulu University Bioethics on 5 June 2025 (WSU HREC 06/2025) and the Eastern Cape Department of Health on 12 June 2025 (EC_202506_011). Participation was voluntary and anonymous. The participants provided informed consent and had the right to withdraw from the study.

## Results

Out of 131 community members surveyed (Table 1), TB knowledge was categorized as low (11.5%), moderate (64.9%), and high (23.7%). Gender did not significantly influence knowledge levels (*p* = 0.5), although women made up 73% of the sample and were slightly more represented in the high knowledge group (81%). The median age was 48 years, with no significant differences across knowledge levels (*p* = 0.2); however, the high knowledge group tended to be younger (median age 43) compared to the low knowledge group (median age 59). Marital status showed a borderline association with knowledge (*p* = 0.054), with single individuals more common in the high knowledge group (71%) than the married group (19%). Nearly all participants identified as Black African (99%) and the majority as Christian (85%), though religion was not significantly associated with knowledge levels (*p* = 0.13). Education level showed a strong and significant association with TB knowledge (*p* < 0.001); notably, 52% of participants with high knowledge had a tertiary education, while none in the low knowledge group had reached this level. Those without formal education were disproportionately represented in the low knowledge group (27%), underscoring the impact of educational attainment on TB awareness. Among the 129 respondents who reported reasons for not testing for TB, the most cited barriers were fear of stigma (42%) and lack of knowledge (33%). Structural factors such as distance to clinics (10%) and cost (7%) were also mentioned. Although these barriers were not significantly associated with knowledge levels (*p* = 0.2), participants with low TB knowledge were more likely to report cost and distance as barriers (both at 21%). TB experience and contact history were found to influence knowledge levels among participants. Overall, only 12% reported having previously had TB, with none in the low knowledge group having personal experience, compared to 16% in the high knowledge group—a statistically significant difference (*p* = 0.047). Additionally, having a close relative who had TB was strongly associated with higher knowledge (*p* < 0.001); 52% of participants in the high knowledge group reported a family history of TB, in contrast to just 6.7% in the low knowledge group.

**Table 1:** Socio-demographic characteristics of participants, including KAP survey.

Characteristic	*N*	Overall *N* = 131^a^	Low *N* = 15^a^	Moderate *N* = 85^a^	High *N* = 31^a^	*p*-value^b^
Gender	131					
Female		95 (73%)	10 (67%)	60 (71%)	25 (81%)	0.5
Male		36 (27%)	5 (33%)	25 (29%)	6 (19%)	
Age	131	48 (30, 60)	59 (36, 75)	44 (30, 60)	43 (31, 57)	0.2
Marital status	131					
Single		71 (54%)	5 (33%)	44 (52%)	22 (71%)	0.054
Married		51 (39%)	9 (60%)	36 (42%)	6 (19%)	
Divorced		2 (1.5%)	0 (0%)	2 (2.4%)	0 (0%)	
Widowed		7 (5.3%)	1 (6.7%)	3 (3.5%)	3 (9.7%)	
Ethnicity	131					
Black		130 (99%)	15 (100%)	84 (99%)	31 (100%)	>0.9
Colored		1 (0.8%)	0 (0%)	1 (1.2%)	0 (0%)	
Religion	131					
Christian		112 (85%)	11 (73%)	72 (85%)	29 (94%)	0.13
Traditional		12 (9.2%)	4 (27%)	7 (8.2%)	1 (3.2%)	
Other		7 (5.3%)	0 (0%)	6 (7.1%)	1 (3.2%)	
Highest education level	131					
None		14 (11%)	4 (27%)	10 (12%)	0 (0%)	<0.001
Primary		33 (25%)	4 (27%)	25 (29%)	4 (13%)	
Secondary		58 (44%)	7 (47%)	40 (47%)	11 (35%)	
Tertiary		26 (20%)	0 (0%)	10 (12%)	16 (52%)	
Reason for not testing for TB	129					
Cost		9 (7.0%)	3 (21%)	5 (6.0%)	1 (3.2%)	0.2
Distance		13 (10%)	3 (21%)	8 (9.5%)	2 (6.5%)	
Fear of stigma		54 (42%)	4 (29%)	38 (45%)	12 (39%)	
Lack of knowledge		42 (33%)	4 (29%)	24 (29%)	14 (45%)	
Other		11 (8.5%)	0 (0%)	9 (11%)	2 (6.5%)	
Ever had TB	131					
Once had TB		16 (12%)	0 (0%)	11 (13%)	5 (16%)	0.047
Never had TB		97 (74%)	9 (60%)	65 (76%)	23 (74%)	
I do not know		18 (14%)	6 (40%)	9 (11%)	3 (9.7%)	
Close relative once had TB	129					
Close relative once had TB		40 (31%)	1 (6.7%)	23 (28%)	16 (52%)	<0.001
Close relative never had TB		74 (57%)	7 (47%)	53 (64%)	14 (45%)	
I do not know		15 (12%)	7 (47%)	7 (8.4%)	1 (3.2%)	

a *n* (%); median (*Q*1, *Q*3).

b Fisher’s exact test; Kruskal–Wallis rank sum test.

**Figure 1:**
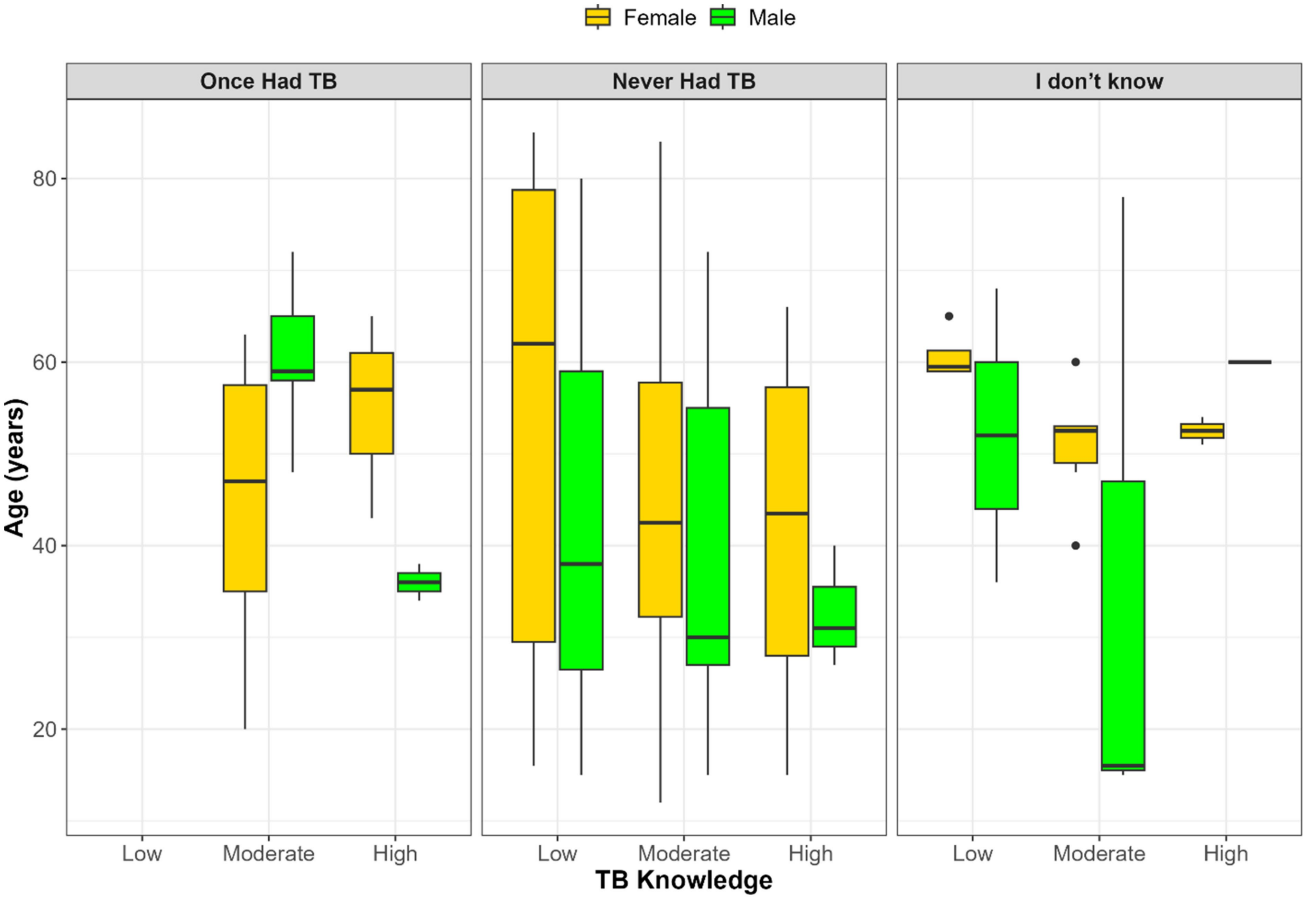
Age and gender distribution across TB knowledge levels stratified by self-reported TB history.

Figure 1 illustrates the intersection between TB knowledge, age, gender, and self-reported TB history. Among participants who had previously had TB, only moderate and high knowledge levels were observed, with older females more prevalent in the moderate group and younger males more common in the high knowledge category. In contrast, those who had never had TB exhibited a broader age distribution, but high TB knowledge remained more frequent among younger males. Participants uncertain of their TB history showed wide variability in age and inconsistent knowledge levels, with minimal representation in the high knowledge group. Overall, high TB knowledge was more commonly observed among younger participants, particularly males, while females were generally older across all knowledge categories.

**Figure 2:**
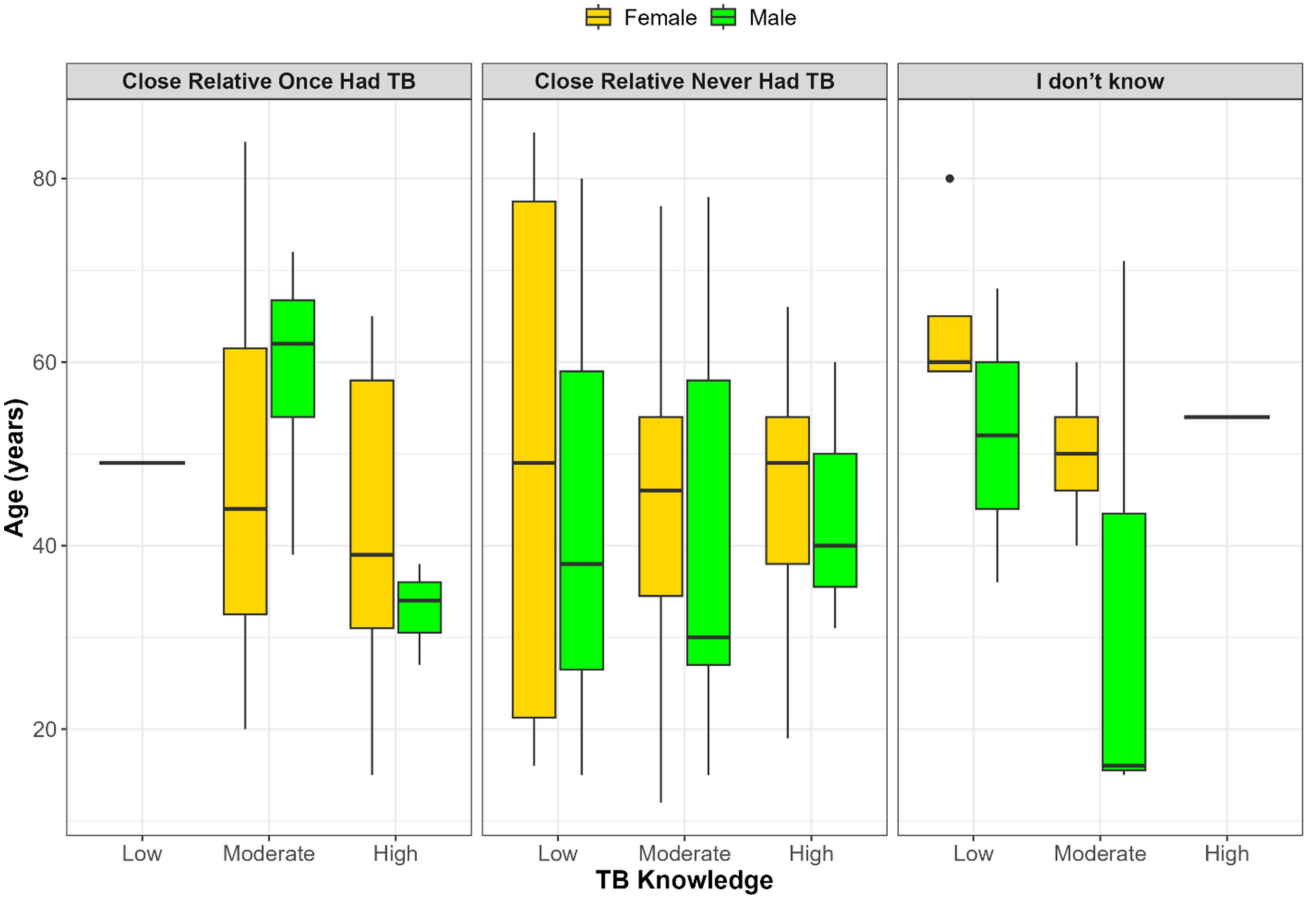
Distribution of age and gender across TB knowledge levels (low, moderate, high) stratified by participants’ knowledge of whether a close relative once had TB.

Figure 2 illustrates the relationship between TB knowledge, age, gender, and whether participants had a close relative who previously had TB. Among those who confirmed a family history of TB, TB knowledge was generally higher, particularly among younger males. In contrast, participants who reported no relative with TB showed greater variation in both age and knowledge levels, with no clear pattern by gender. The smallest group, those uncertain of their family TB history, had inconsistent age and knowledge distributions, with sparse representation in the high knowledge category. Additionally, age and gender disparities persist, with older females more common in lower knowledge categories and younger males more frequently appearing in higher knowledge groups.

## Discussion

This pilot study in rural Ntabankulu reveals that a substantial proportion of the community has low (11.5%) or moderate (64.9%) knowledge of tuberculosis (TB), with only 23.7% demonstrating high knowledge. These findings are consistent with similar surveys in rural South Africa, which identify limited TB literacy as a persistent barrier to effective prevention and care in underserved regions (16–18). The findings from this study are, however, inconsistent with those from other studies conducted in Limpopo Province, Ethiopia, and India, where a majority of participants reported high TB knowledge (75%, 94.9%, and 94%, respectively) (19–21). This variation likely stems from differences in study populations, time periods, and settings (rural versus urban), as well as the unique characteristics of the local health system. Factors such as Ntabankulu’s remoteness and limited access to primary healthcare facilities and community health workers could also contribute to the lower knowledge levels. Given that TB is highly contagious and has a high burden in South Africa, especially in the rural Eastern Cape, communities must understand the signs and symptoms to curb transmission. The findings highlight an urgent need for continuous educational interventions to raise awareness of the disease in these rural areas.

### Education and knowledge correlation

Educational attainment was the strongest predictor of TB knowledge (*p* < 0.001). None of the participants in the low knowledge group had a tertiary education, while over half (52%) of those with high knowledge had completed tertiary studies. These findings align with previous research, including a study in KwaZulu-Natal that found a strong correlation between health literacy and formal education (22). Similarly, a cross-sectional survey in Indonesia reported a significant association (*p* < 0.001) between education level and knowledge of pulmonary TB, with highly educated individuals demonstrating greater awareness (23). Ma et al. (24) also found a positive relationship between higher education and improved TB knowledge and practices.

The results underscore the need for tailored health education strategies that accommodate various literacy levels, particularly for populations with limited formal education. These strategies should employ culturally relevant, visual, and spoken communication methods to be most effective.

### Barriers to TB testing

The findings reveal that the most common barriers to TB testing were fear of stigma (42%) and lack of knowledge (33%). Although structural challenges like distance (10%) and cost (7%) were less prominent, they were disproportionately cited by individuals with low knowledge. This suggests that misinformation may worsen perceived access issues. While a direct statistical association between these barriers and knowledge levels was not found (*p* = 0.2), participants with low TB knowledge were more likely to report cost and distance as barriers (both at 21%). This highlights the combined burden of psychosocial and structural obstacles in rural settings, where limited knowledge can compound the perception and experience of access-related challenges.

These results are consistent with other studies that have identified stigma as a primary deterrent to early testing (25, 26). Global literature also points to stigma, misinformation, and low health literacy as major barriers to TB care in rural communities (4, 10).

A key implication of this study is the need for structured, participatory community engagement (CE) models. Successful models in Vietnam and Mexico have demonstrated that community-driven approaches—involving TB survivors, peer educators, and local structures—can significantly improve early case detection and treatment adherence (7, 8). For example, Ethiopia’s participatory action framework, which involved stakeholders and community leaders, improved TB elimination efforts by aligning health interventions with local needs (5). Similarly, the Peru-based TB Móvil project demonstrated how social networks and local champions could transform health education into a community-driven movement (9).

Applying these models to the Eastern Cape could involve:

Engaging TB survivors as “treatment ambassadors.”

Establishing local advisory boards.

Training community health workers (CHWs) in culturally relevant messaging.

Strengthening collaborations between the health system and traditional or civic leaders may also enhance program legitimacy and reach. Furthermore, capacity-building initiatives like CE training and youth engagement programmes can help address knowledge gaps, especially among younger populations. Ensuring that community voices are part of the process, from planning to evaluation, is essential for building trust and avoiding tokenism (27).

### TB experience and contact history

No statistically significant association was found between gender, age, and TB knowledge (*p* > 0.05). Boxplot visualizations suggested possible differences, such as younger males appearing more frequently in higher knowledge groups and older females in lower categories. However, these patterns should be interpreted cautiously as exploratory observations rather than firm conclusions, given their lack of statistical significance and the limited sample size of this pilot. Larger, more representative studies are needed to determine whether such demographic differences truly exist or whether they reflect artifacts of sampling and small subgroup counts.

These findings are consistent with previous studies, which have shown that lived experiences and peer-to-peer education enhance community TB knowledge (28–30). A study in India, for example, found that individuals with a personal or family history of TB demonstrated substantially better knowledge of infection prevention and treatment protocols (31).

This underscores the value of leveraging the experiences of survivors and their families in community-based interventions. Peer educators who share their lived experiences can act as trusted communicators, helping to not only increase knowledge but also to combat misinformation and stigma. They can also serve as a crucial link between communities and the health system, assisting individuals in seeking and navigating complex care pathways (32).

### Sociodemographic patterns: age and gender

While no statistical association was found between gender, age, and TB knowledge, there was a noticeable trend: younger males tended to dominate the high-knowledge group, while older females were more prevalent in the moderate and low-knowledge categories. These findings align with some prior research showing no relationship between age and TB knowledge (33). However, they contrast with other studies that found a significant association between female gender and higher knowledge (19, 34, 35). This difference may be attributed to the traditional caregiving roles of women, which often increase their interaction with healthcare services and exposure to health information (34). The intersection of age, gender, and TB history suggests that knowledge is shaped by these factors. This highlights the importance of targeted educational strategies that consider these specific demographic patterns.

### Comparison to urban settings

The lower TB knowledge levels observed in this rural community, compared to urban areas, reflect broader rural–urban health disparities. In northeast Tanzania, for instance, rural adults had significantly lower TB knowledge than their urban counterparts (12). Similar disparities have been reported in Lesotho, Nigeria, and Ethiopia, often linked to differences in educational levels and access to healthcare facilities (36–38). Urban areas benefit from widespread clinic-based and mass media campaigns, whereas rural populations often lack such access, leading to significant knowledge gaps. This disparity underscores the urgent need for context-specific and community-led interventions in rural areas. Strategies should focus on reducing stigma, improving access, and using locally relevant educational approaches to create equitable TB care (39).

### Persistent knowledge gaps and structural barriers

First, the predominance of *low to moderate knowledge* (76.4% of participants) reflects persistent gaps in community awareness that hinder early diagnosis and effective treatment uptake. Similar knowledge deficiencies have been documented in rural populations across Sub-Saharan Africa, where TB literacy remains uneven due to structural barriers, limited access to health promotion, and entrenched *stigma* (39, 40, 41).

### Socioeconomic determinants of health literacy

The strong association observed between *educational attainment and TB knowledge* underscores the role of socioeconomic determinants in shaping health awareness. Individuals with tertiary education demonstrated significantly greater TB knowledge, while those with little or no formal education were more likely to fall into the low-knowledge category. This gradient highlights the need for communication strategies that extend beyond written materials to include *oral, visual, and community-based approaches* tailored to populations with limited literacy.

### The critical role of lived experience

Our findings also point to the critical role of lived experience. Both *personal and familial TB history* were strongly linked to higher knowledge scores, suggesting that TB survivors and their families can serve as powerful *peer educators* within their communities. This finding aligns with global literature showing that survivor-led education and participatory interventions can reduce stigma, improve adherence, and promote timely health-seeking (10).

### Limitations

While this pilot study provides valuable insights into TB knowledge and barriers in a rural South African community, the findings should be interpreted with caution. The small sample size (*n* = 131) limited the statistical power to detect associations, which may explain why some variables, such as gender and age, showed trends but did not reach statistical significance. Similarly, the borderline significance observed for marital status may be due to low subgroup counts rather than the absence of a true relationship.

The use of convenience sampling, while practical for a pilot study, may have introduced selection bias. Participants were recruited from a community outreach event, which could over-represent individuals who are already more health-seeking or engaged with health services. This may partly explain the relatively high levels of moderate knowledge observed, compared to studies in other underserved populations. Conversely, the underrepresentation of individuals who are too unwell or unable to attend the outreach may have masked the true extent of knowledge deficits and barriers in the wider community. These limitations do not diminish the importance of the findings but rather highlight the need for larger, more representative studies. Such studies could clarify whether the associations seen here, for example, the link between TB knowledge and education or prior TB exposure, hold consistently across different subpopulations. They would also help disentangle whether weak or inconsistent associations observed in this pilot reflect real community patterns or methodological constraints.

### Recommendations

Based on the findings from this pilot study, particularly the strong influence of education and prior TB experience on TB knowledge, and the prominence of stigma and misinformation as barriers to testing, we make the following locally derived recommendations. Where appropriate, we also highlight strategies from international literature that could complement these local findings. Develop culturally appropriate educational materials: Create low-literacy materials in local languages, such as isiXhosa, using visual aids, community radio, and storytelling to effectively reach those with limited formal education. Integrate TB survivors and families: Involve individuals with lived experience in peer education and community mobilization to reduce stigma and build public trust. Strengthen community health worker (CHW) training: Equip CHWs with skills in stigma-sensitive communication to deliver household-level TB education and screening.

Expand mobile TB screening services: Address persistent structural barriers, such as distance and cost, by expanding mobile TB screening to remote and underserved areas. Conduct larger studies: Perform larger, multi-site studies to validate these pilot findings and better understand regional variations in TB knowledge, stigma, and engagement patterns, which will inform more targeted public health interventions.

## Conclusion

This pilot study reveals critical gaps in TB knowledge and the complex interplay between education, stigma, and lived experience in the rural Eastern Cape. While some awareness exists, a significant portion of the population remains underserved with accurate TB information, particularly individuals with limited formal education or familial TB exposure. Psychosocial barriers, such as stigma, persist alongside structural challenges, impeding early diagnosis and care-seeking. These findings suggest that TB elimination in rural South Africa is unlikely without deeply rooted community engagement. Addressing these challenges requires community-driven, inclusive, and culturally appropriate education strategies. Such interventions should be embedded within primary healthcare and supported by strong clinical governance. By leveraging local knowledge, co-designing educational interventions, and empowering residents as agents of change, rural communities can be empowered to play an active role in TB prevention and contribute to South Africa’s broader TB elimination goals.

## Data Availability

The raw data supporting the conclusions of this article will be made available by the authors, without undue reservation.

## Walter Sisulu University TB Research Group Medical Microbiology 2025 Honours Students

Bulela Sonka, Hloniphani Guma, Londiwe Nxumalo, Luvo Ntombana, Luzuko Mkono, Mandlenkosi Manika, Mbulelo Cebisa, Muziwabantu Joyi, Nande Ndamase, Phumelela Bana, Siphosihle Conham, Sithabile Moso, Yanga Somjobo.
