# Correction: Towards community-driven tuberculosis education: findings from a knowledge and engagement pilot survey in the rural community of Eastern Cape, South Africa

**DOI:** 10.3389/fpubh.2025.1756648

**Published:** 2026-01-08

**Authors:** Ntandazo Dlatu, Urgent Tsuro, Lindiwe Modest Faye, Mojisola Clara Hosu, Ncomeka Sineke, Teke Apalata

**Affiliations:** 1 Department of Public Health, Faculty of Medicine and Health Sciences, Walter Sisulu University, Mthatha, South Africa; 2 Division of Medical Microbiology, Department of Laboratory-Medicine and Pathology, Faculty of Health Sciences, Walter Sisulu University, Mthatha, South Africa; 3 Department of Laboratory Medicine and Pathology, Faculty of Medicine and Health Sciences, Walter Sisulu University, Mthatha, South Africa

**Keywords:** tuberculosis (TB), TB knowledge, stigma, barriers to testing, rural South Africa, Eastern Cape, health education, KAP survey

The title of this article was erroneously given as: “Toward community-driven tuberculosis education: findings from a knowledge and engagement pilot survey in the rural community of Eastern Cape, South Africa” The correct title of the article is “Towards community-driven tuberculosis education: findings from a knowledge and engagement pilot survey in the rural community of Eastern Cape, South Africa”

The **Author contributions** statement has been corrected to include the full initials for authors Lindiwe Modest Faye and Mojisola Clara Hosu. They are now written as “LMF” and “MCH”.

The collaborators in the Walter Sisulu University TB Research Group Medical Microbiology 2025 Honours Students were not listed in an end statement. A correction has been made to include their names in an end statement as follows:

## Walter Sisulu University TB Research Group Medical Microbiology 2025 Honours Students

Bulela Sonka, Hloniphani Guma, Londiwe Nxumalo, Luvo Ntombana, Luzuko Mkono, Mandlenkosi Manika, Mbulelo Cebisa, Muziwabantu Joyi, Nande Ndamase, Phumelela Bana, Siphosihle Conham, Sithabile Moso, Yanga Somjobo.

Author Urgent Tsuro was erroneously assigned to affiliation “Department of Public Health, Faculty of Medicine and Health Sciences, Walter Sisulu University, Mthatha, South Africa.” This affiliation has now been removed for author Urgent Tsuro.

Affiliation “Division of Medical Microbiology, Department of Laboratory-Medicine & Pathology, Faculty of Health Sciences, Walter Sisulu University, Mthatha, South Africa” was omitted for author Urgent Tsuro. This affiliation has now been added for author Urgent Tsuro.

“Author Ncomeka Sineke was erroneously spelt as Sineke Ncomeka” accurately reflects this correction. We also agree with the updated author citation as “Sineke N” and the use of “NS” for the author contributions statement.

The original version of this article has been updated.

